# Response: Commentary: Aesthetic Pleasure versus Aesthetic Interest: The Two Routes to Aesthetic Liking

**DOI:** 10.3389/fpsyg.2018.00472

**Published:** 2018-04-20

**Authors:** Laura K. M. Graf

**Affiliations:** Chair for Product Management and Marketing Communications, Goethe University Frankfurt, Frankfurt am Main, Germany

**Keywords:** pleasure, interest, aesthetic liking, processing fluency, processing style

In his commentary on the paper “Aesthetic Pleasure versus Aesthetic Interest: The Two Routes to Aesthetic Liking,” authored by Jan R. Landwehr and myself (Graf and Landwehr, 2017), Consoli (2017) deplores two aspects of our paper. First, an inadequate definition and operationalization of the key constructs aesthetic pleasure, aesthetic interest, and aesthetic liking, respectively aesthetic attractiveness. Second, the conclusions drawn from our empirical studies. While I acknowledge that one may have a different theoretical perspective on aesthetic perception and evaluation, it appears that Consoli's (2017) commentary does not even address the empirical data of our studies but only our theoretical assumptions and definitions. In the following, I will address Consoli's (2016, 2017) arguments in more detail, and I will corroborate our theoretical reasoning with the empirical data of our studies (Graf and Landwehr, 2017).

Consoli (2016, 2017) argues that interest represents a pre-insight anticipation evoked only by the expectation of coping potential (not by a post-insight reaction), and that pleasure has a “twofold affective nature” including both a pre-insight and a post-insight affective reaction. However, bearing in mind that we construe aesthetic interest as an evaluative aesthetic response, this would imply that interest evaluations can occur without a previous processing experience with the stimulus. In our view, this does not make sense; how else than engaging with the aesthetic object should people assess that they find the object interesting? As detailed in our Pleasure-Interest Model of Aesthetic Liking (PIA Model; Graf and Landwehr, 2015), it is much more reasonable that an evaluative aesthetic interest response is informed by the processing experience and thus also entails a post-processing component. More specifically, it is the processing experience of disfluency reduction which triggers aesthetic interest: disfluency reduction indicates that by investing cognitive energy into processing the stimulus, one can learn new things and that the stimulus is therefore interesting. Hence, in contrast to Consoli's (2017) perspective, according to which interest requires only the expectation of coping potential, disfluency reduction in the PIA Model (Graf and Landwehr, 2015) represents an actual experience of successful coping.

Moreover, Consoli (2016, 2017) does not consider the consequences of the duality of processing into automatic and controlled processing with regard to the conceptualizations of pleasure and interest. As explained in the PIA Model (Graf and Landwehr, 2015), processing fluency, or, as Consoli (2017) puts it, a “post-insight affective reaction” is interpreted differently when it occurred during automatic processing compared to when it occurred during controlled processing (Graf and Landwehr, 2015). Specifically, when people process an aesthetic stimulus automatically, the post-insight affective feeling informs the perceiver mainly about the ease or difficulty of processing the visual characteristics of a stimulus and thus triggers an aesthetic pleasure response. However, when people process an aesthetic stimulus controlled, the post-insight affective reaction (which is disfluency reduction in the PIA Model) is the result of the investment of cognitive effort and thus informs the perceiver also about his or her ability to handle the stimulus and to learn something from the stimulus. Thus, people will rather evaluate the stimulus as interesting as opposed to simply pleasing (see also Thesis 1 of the PIA Model; Graf and Landwehr, 2015).

So far, the existing empirical results clearly support our conceptualization of interest, showing that interest is positively influenced by disfluency reduction and hence by a post-insight affective reaction (Study 1; Graf and Landwehr, 2017). Moreover, additional analyses show that even though the effect of stimulus fluency on pleasure is weekly mediated by disfluency reduction, this mediation effect is less pronounced under a controlled processing style. Importantly, this suggests that the association between disfluency reduction and pleasure differs from the association between disfluency reduction and interest, and that pleasure is the less appropriate reaction following disfluency reduction under controlled processing.

Presumably, Consoli (2017) overlooked the most central assumption of the PIA Model and hence the theoretical foundation of our empirical studies (Graf and Landwehr, 2015, 2017). Specifically, he argues that our conceptualization of an aesthetic liking response, respectively an overall aesthetic preference judgment, is not in accordance with the established notion that automatic and controlled processes produce different outcomes. However, this exactly is the main motivation of our research; we argue that aesthetic preferences that accrue from automatic versus controlled processing approaches are fundamentally different, and that generic preference judgments such as liking obscure these particularities. In addition, this is also the main reason why the post-insight affective reaction under controlled processing is better reflected by interest evaluations than by pleasure evaluations (Graf and Landwehr, 2015).

Most importantly, Consoli (2017) conceptualizes pleasure and interest at different levels within the aesthetic preference formation process. Specifically, whereas we conceptualize aesthetic pleasure and interest as evaluative outcomes of the aesthetic preference formation process (Graf and Landwehr, 2015, 2017), Consoli (2016, 2017) construes them as higher-order affective signals within the dynamic aesthetic preference formation process itself. Put differently, Consoli's (2016, 2017) understanding of the functions of pleasure and interest correspond to our understanding of the experiences of fluency and disfluency reduction.

In summary, given Consoli's (2016, 2017) fundamentally different theoretical perspective, it comes as no surprise that he does not agree with our conceptualizations of aesthetic pleasure, interest, and liking. Even though our empirical data clearly favor our theoretical approach (Graf and Landwehr, 2015), I believe that it would be interesting to combine Consoli's (2016, 2017) theoretical approach with ours, for instance by applying his idea of anticipation and reaction to processing fluency, for an even richer understanding of the aesthetic preference formation process.

## Author contributions

The author confirms being the sole contributor of this work and approved it for publication.

### Conflict of interest statement

The author declares that the research was conducted in the absence of any commercial or financial relationships that could be construed as a potential conflict of interest.
